# 2236. Evaluation Of Antibiotic Utilization For Urinary Tract Infections In Two Community Hospitals In Miami

**DOI:** 10.1093/ofid/ofad500.1858

**Published:** 2023-11-27

**Authors:** Renata Boatwright, Veronica Salazar, Elianis Quintana, Rossana Rosa, Lilian M Abbo

**Affiliations:** Jackson South Medical Center, Miami, Florida; Jackson Memorial Hospital, Miami, FL; Nova Southeastern University, Miami, Florida; Jackson Memorial Hospital, Miami, FL; University of Miami Miller School of Medicine, Miami Transplant Institute and Jackson Health System, Miami, FL

## Abstract

**Background:**

Antimicrobial Stewardship (ASP) of urinary tract infections (UTIs) remain a challenge as UTIs continue to be over-diagnosed. Lack of documentation of symptoms, unclear clinical indication for cultures and improper culture collection are some of the contributing factors. Thus, over-diagnosis of UTIs leads to unnecessary antimicrobial exposures adding to increased risk of *Clostridioides difficile* infection and antimicrobial resistance. Our study aimed at assessing the magnitude of inappropriate antimicrobial exposures related to UTIs at Jackson South (JSMC) and Jackson North (JNMC) Medical Centers, two mid-size hospitals in the Miami area, to guide future stewardship measures.

**Methods:**

A retrospective chart review of 18% of UTI orders during March 2023 was conducted. The orders were evaluated for four criteria: documentation of UTI symptoms; antimicrobial selection, dose and frequency; treatment duration including antibiotic prescriptions upon discharge and appropriateness of testing. Antibiotic orders were deemed appropriate if all criteria were met. Prescribing pattern was also evaluated for critical care status.

**Results:**

A total of 3,472 antibiotic orders were prescribed during the study period of which 10% were indicated for UTI. The study was powered at 95% confidence interval. Inappropriate testing was the most common occurrence at 42%, followed by treatment duration 32%. JNMC prescription orders contributed to 72% of inappropriate treatment length compared to 40% at JSMC. Lack of UTI symptom documentation represented 28% of the cases and lastly antibiotic choice, dose and frequency contributed with 16%. Overall, 36% of the UTI orders were deemed appropriate at JSMC versus 20% at JNMC (Fig. 01).The majority of the orders were outside of critical care units for both centers (Fig. 02).

**Figure:**
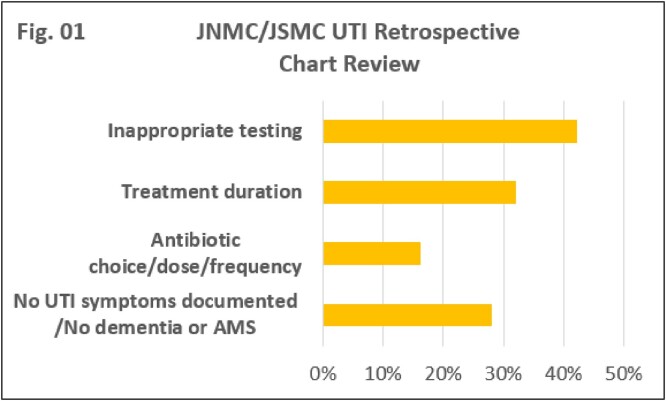

**Figure:**
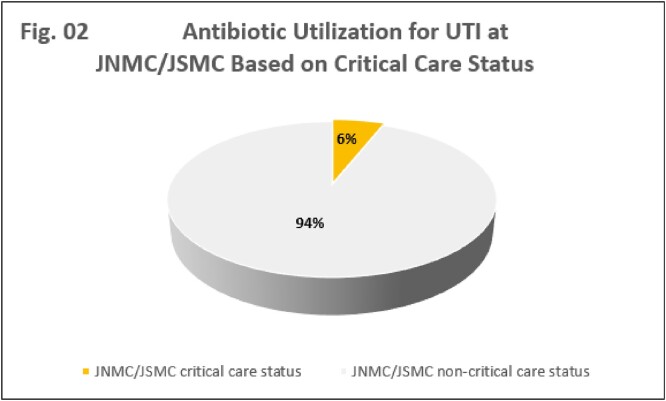

**Conclusion:**

UTI over-diagnosis has led to inappropriate antibiotic usage. Excessive laboratory testing, prolonged treatment duration and lack of assessment of UTI symptoms were the main opportunities identified. Leveraging algorithm technology for increased compliance with symptom assessment and reduced screening as well as implementation of outpatient prescription stewardship would be beneficial to the health system.

**Disclosures:**

**Lilian M. Abbo, MD, MBA**, Ferring: Advisor/Consultant|Pfizer: Advisor/Consultant|Regeneron: Grant/Research Support|Shionogi: Advisor/Consultant

